# Hydrothermal Synthesis of ZnO Superstructures with Controlled Morphology via Temperature and pH Optimization

**DOI:** 10.3390/ma16041641

**Published:** 2023-02-16

**Authors:** Aleksander Ejsmont, Joanna Goscianska

**Affiliations:** Faculty of Chemistry, Department of Chemical Technology, Adam Mickiewicz University, Uniwersytetu Poznańskiego 8, 61-614 Poznań, Poland

**Keywords:** zinc oxide, particles shape, citric acid, capping agent, morphology control, tunable microstructures

## Abstract

Zinc oxide, as a widely used material in optics, electronics, and medicine, requires a complete overview of different conditions for facile and easily reproducible syntheses. Two types of optimization of ZnO hydrothermal preparation from zinc acetate and sodium hydroxide solution are presented, which allowed for obtaining miscellaneous morphologies of materials. The first was a temperature-controlled synthesis from 100 to 200 °C, using citric acid as a capping agent. The formation of hexagonal rods at the lowest temperature was evidenced, which agglomerated to flower-like structures at 110 and 120 °C. It was followed by transformation to flake-like roses at 160 °C, up to disordered structures composed of nanosized plates (>180 °C). The transformations were generated through a temperature change, which had an impact on the diffusion effect of hydroxide and citrate complexes. The second optimization was the hydrothermal synthesis free of organic additives and it included only a pH variation from 7.5 to 13.5. It was found that by utilizing a slow-dropping process and varying amounts of NaOH solutions, it is possible to obtain well-formed hexagonal pellets at pH 8.0–8.5. Strongly basic conditions of pH 11.0 and 13.5 impeded superstructure formations, giving small elongated particles of ZnO. All samples were characterized by high phase purity and crystallinity, with a specific surface area of 18–37 m^2^/g, whereas particle size distribution indicated a predominance of small particles (<1 μm).

## 1. Introduction

Innovative synthetic solutions for complex materials are steadily pursued, leaving behind those that seem to be well-established. The implementation of emerging materials usually involves time and effort, while there is still ample room for exploration and improvement of materials currently in use. Such an example is zinc oxide, broadly utilized in industrial, technical, and medical applications [1]. The great technological importance of ZnO is due to its lack of a symmetry center (wurtzite structure), high electromechanical coupling (piezo-/pyroelectric properties), wide band gap (3.37 eV), high exciton binding energy (60 meV), antibacterial efficacy, and more [2,3,4,5,6,7]. In addition, the ZnO crystals exhibit partial polar characteristics [8]. Its atomic structure results in a normal dipole moment, polarization along the *c*-axis, and disparities in surface energy. However, ZnO is a highly appealing material in terms of morphology. Based on its structure, it possesses three types of directions for rapid growth. Among the innermost factors governing the morphology is the relative surface activity of different growth surfaces under specific conditions. The crystal exhibits different kinetic parameters for distinct crystal planes, which are accentuated in regulated growing regimes [9]. By optimizing the synthesis conditions, it is possible to directly influence the final shape of the material, and demonstrate the impact of reaction factors on its formation.

Morphology is defined as the shape, texture, or surface topography of a material. One general classification of materials is based on their dimensionality, i.e., one, two, or three-dimensional, for example, for dots, threads, and branches, respectively [10]. An important point is that ZnO particles can exhibit diverse shapes, and this in turn can contribute to its different macroscopic properties determining various applications [11,12]. So far, ZnO has been obtained in many forms, e.g., rods, platelets, flowers, belts, flakes, and films [13]. ZnO microflowers showed potential in photocatalytic degradation of dyes [14], ZnO nanorods had an impact on long-term stability of Perovskite Solar Cells [15], and microrods revealed utility as photodetectors [16]. Equally diversified are the methods of ZnO preparation, such as sol-gel, controlled precipitation, and solvothermal syntheses, or those utilizing emulsions, mechanochemistry, or biological extracts [1,17,18]. Mishra et al. proposed the synthesis of ZnO tetrapods [19]. In the flame transport synthesis, they optimized the nanoarms’ shape, which was influenced by the growth time (30–90 min) and temperature (900–950 °C). As a result, it led to the tetra- and multipods formation. Moreover, the materials obtained revealed multifunctionality and were applied in photocatalysis, UV photodetection, and gas sensing [19]. Ayyub and co-workers reported an electrodeposition method in which by changing the potential and electrode separation they influenced the diameter of ZnO nanorods. They were able to produce particles with diameters such as 104, 194, 205, 266, and 485 nm [20].

Among the various ZnO fabrication techniques, the hydro-/solvothermal method is one of the main routes of synthesizing unique ZnO shapes, due to the possibility of rigorous control over the growth of crystallites but also relatively facile up-scaling. Four different parameters can influence the crystallization at non-equilibrium kinetic growth conditions: kinetic barrier, capping molecules, time, and temperature [21]. For instance, by using the appropriate temperature or pH of the reaction, the kinetic barrier can be overcome faster and the rate of crystallization can be regulated. The very course of the synthesis can be also affected by the choice of salt as the metal source for the metal oxide. Acetate salts are often selected for the synthesis of unique nanomaterials due to better diffusion of metal cations in aqueous solutions, which contributes to the more frequent preparation of homogeneous and smaller particles [22]. Moreover, by changing the polarity of solvents, as well as by using capping agents or surfactants, one can support shifting the surface activity of the crystallite and force crystallization in a specific direction. However, for this purpose, high-cost organic reactants are often applied, which additionally contradict the idea of green chemistry. Therefore, it is important to optimize syntheses in aqueous solutions using non-toxic, inexpensive, and safe reactants.

Up to now, it has been established that the presence of capping agents can contribute to the creation of flower-like structures [21,23,24,25,26]. The syntheses of ZnO using different concentrations of citric acid (CA) [23], and a wide range of its derivatives (e.g., triethyl citrate, tripotassium citrate, trisodium citrate, and triammonium citrate) have been reported [27]. Due to utilizing auxiliary reactants, it was possible to yield varying ZnO particle shapes resembling small, thick, or elongated flowers. However, to the best of our knowledge, the influence of various temperatures at a constant concentration of CA on the ZnO morphology has not been considered. Hence, the present work aimed to optimize the hydrothermal synthesis of ZnO in the presence of citric acid (CA), in which the reaction temperature was changed in the range of 100–200 °C. As a result, particles of different morphologies were obtained. Moreover, after determining the most favorable reaction temperature for our system, the synthesis free of organic additives was carried out with altered pH from 7.5 to 13.5. Its optimization was designed to establish the most favorable conditions for the preparation of short hexagonal pellets (dog-bones) of ZnO, which in previous reports have been obtained using, e.g., tetraethylene glycol [28] or triethylamine [29]. The possible course of zinc oxide crystallization and its particle agglomeration is proposed. The straightforward and efficient syntheses of ZnO with unique morphologies are in line with the current notion of green chemistry.

## 2. Materials and Methods

### 2.1. Materials

Zinc acetate dihydrate (99.9%, Pol-Aura, Morąg, Poland), citric acid monohydrate (99.9%, POCH, Gliwice, Poland), sodium hydroxide (≥98%, Stanlab, Lublin, Poland).

### 2.2. Temperature-Altered Hydrothermal Synthesis of ZnO

The hydrothermal method was used to obtain zinc oxide particles. First, 4.4 g of zinc acetate dihydrate was dissolved in 1320 mL of distilled water and stirred until completely dissolved. Then, 2.2 g of citric acid was added to the solution and stirred for another 10 min. Then 440 mL of 1 mol L^−1^ sodium hydroxide was slowly dripped in. Once all of it had been instilled, the reaction mixture was transferred to a 2 L Teflon-lined steel autoclave and hydrothermally treated for 24 h at 100, 110, 120, 160, 180, and 200 °C. The products were filtered and washed with water and ethanol three times. The resulting materials were dried in an oven at 60 °C for 24 h. According to the applied temperature, samples were labelled as follows: ZnO_100_, ZnO_110_, ZnO_120_, ZnO_160_, ZnO_180_, and ZnO_200_.

### 2.3. pH-Altered Hydrothermal Synthesis of ZnO

In the first step of zinc oxide synthesis, 30 g of zinc acetate dihydrate was added to 200 mL of distilled water and stirred until completely dissolved. Next, a 1 mol L^−1^ sodium hydroxide solution was prepared and dropped (at a rate of 20 drops/1 min) into the zinc acetate solution. The mixture was stirred vigorously. The pH value was controlled with a pH-meter. Using the same volume of alkali solution as in the temperature-optimized synthesis, a pH of ~13.5 was obtained. For subsequent reaction solutions, the pH was successively adjusted to 11.0, 9.0, 8.5, 8.0, and 7.5 by lowering the volume of the infused sodium hydroxide solution. All six solutions with different pH were transferred to Teflon-lined steel autoclaves and subjected to hydrothermal treatment at 160 °C for 12 h. The resulting products were filtered, washed with distilled water and ethanol, and then dried in an oven at 60 °C for 24 h. Referring to the adjusted pH values, the samples were designated accordingly: ZnO_7.5_, ZnO_8.0_, ZnO_8.5_, ZnO_9.0_, ZnO_11.0_, and ZnO_13.5_.

### 2.4. Samples Characterization

#### 2.4.1. X-ray Diffraction

To confirm the acquisition of a crystalline phase attributed to zinc oxide, an XRD analysis of the samples was performed. XRD patterns were made at room temperature with a step size of 0.05° in the angular range of 10–70° in 2θ, using a D8 Advance Diffractometer (Bruker, Germany) with the copper K_α1_ radiation (*λ* = 1.5406 Å). The crystallite size of the materials obtained in the vertical direction of the corresponding lattice plane (1 0 1) was estimated via Scherrer’s equation (Equation (1)) with Scherrer’s constant equal to 0.9:

(1) $$D=\frac{K\lambda }{\beta \cos\theta }$$

where *D* is the crystallite size, *K* is a shape factor, *λ* is the X-ray wavelength (nm), *β* is the full width at half maximum (FWHM) in radians, and *θ* is the Bragg angle [30].

#### 2.4.2. Scanning Electron Microscopy

To determine zinc oxide particles morphology, scanning electron microscope FEI Helios NanoLab 660 (U.S.) was used. The apparatus was equipped with secondary electron detectors: the Everhart-Thornley Detector (ETD) and Through-Lens Detector (TLD). Before measurements, samples were dispersed in ethanol via sonication and then transferred onto carbon films fixed on an aluminum sample holder, followed by drying in air. The measurements were carried out in the high-vacuum mode (7 × 10^−4^ Pa; RT), in field-free (FF) and high-resolution (HR) modes. The acceleration voltage was 10 kV and the beam current was 0.2 nA.

#### 2.4.3. Fourier-Transform Infrared Spectroscopy

The materials synthesized with the use of citric acid were additionally analyzed in terms of establishing organic residue and surface functional groups, thus their infrared spectra were recorded. The measurements were performed using FT-IR Bruker IFS 66v/S spectrometer (Germany). ZnO powder samples were studied in the form of tablets, previously mixed and pressed with anhydrous KBr with a mass ratio of 1:200 mg. The measuring conditions were as follows, wavenumber range: 4000–400 cm^−1^, resolution: 0.5 cm^−1^, and the number of scans: 64.

#### 2.4.4. Laser Diffraction

ZnO particle size distributions in the range of 0.01–1000 μm were determined with the use of Mastersizer 3000 (Malvern Instruments Co., Ltd., UK). The sample preparation was carried out in a wet unit (Hydro EV) in which a powder material was dispersed in distilled water. The results of the analysis provided the following parameters:

- D[3.2]—Sauter mean diameter, which is an average of particle size;
- D[4.3]—De Brouckere mean diameter, which is a volume mean diameter;
- *d*(0.1)—10% of the particles have diameters smaller than the specified value;
- *d*(0.5)—a median of particle distribution, i.e., 50% of the particles have higher and another 50% of the particles have lower diameters than this value;
- *d*(0.9)—90% of the particles have diameters smaller than the specified value.

#### 2.4.5. Low-Temperature Nitrogen Physisorption

To assess the textural parameters of materials obtained, low-temperature (−196 °C) nitrogen adsorption/desorption was carried out using Quantachrome Autosorb IQ apparatus (U.S.). Before the measurement, samples were degassed at 150 °C for 12 h. The specific surface area (S_BET_) was established based on the Brunauer–Emmet–Teller (BET) method. Whereas average pore diameter and total pore volume were calculated from the adsorption branch of the isotherms using the corrected algorithm of the BJH method (Barrett, Joyner, Halenda).

## 3. Results and Discussion

### 3.1. Characterization of ZnO Particles Obtained via Temperature-Altered Hydrothermal Method

**Figure 1:**
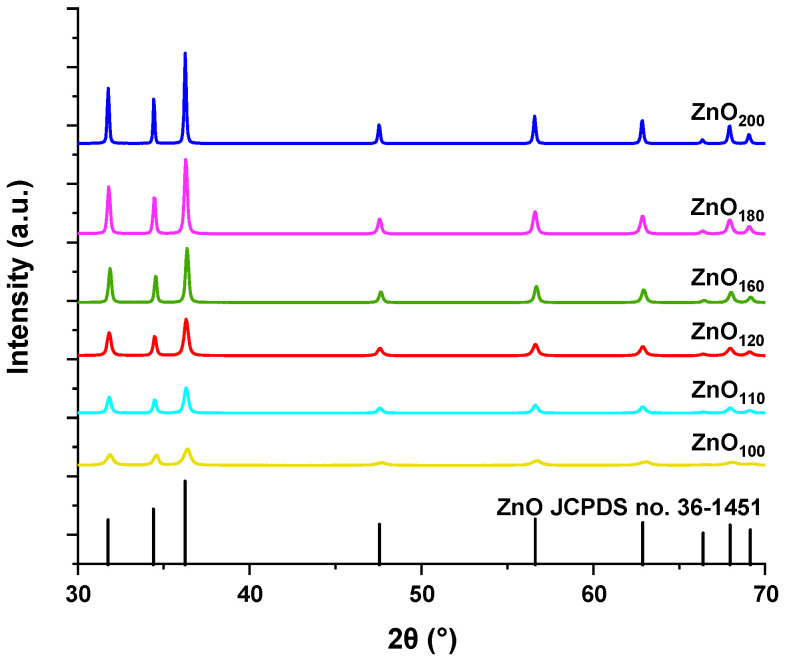
X-ray diffraction patterns of ZnO particles synthesized hydrothermally at 100, 110, 120, 160, 180, and 200 °C. All compared to the ZnO standard, polycrystalline hexagonal wurtzite (JCPDS card No. 36-1451).

The results of the XRD analysis are presented in Figure 1. They confirmed the acquisition of crystalline phases typical of zinc oxide (wurtzite) with hexagonal structure and space group *C6mc*. The reflections featured in the diffractograms can be sequentially assigned to (100), (002), (101), (102), (110), (103), and (112) planes. They are consistent with the polycrystalline ZnO standard (JCPDS card No. 36-1451) [31]. All materials showed high phase purity as well as crystallinity. However, they differ in the degree of crystallinity, which can be inferred by the intensity of the peaks. The reflections’ intensity increases with a higher temperature of synthesis. It was established that the ZnO_100_ sample is the least crystalline, whereas the sample synthesized at 200 °C is characterized by the highest crystallinity. Depending on the synthesis temperature, the size of the zinc oxide crystallites was in the range of 16.80–56.15 nm (Table 1). The effect of the calcination temperature (450–750 °C) on the linear growth of crystallites (38–50 nm) was also previously reported [32]. A similar trend occurred in hydrothermal synthesis at temperatures of 125–200 °C using microwaves, which led to a smaller size of the crystallites from 4 nm to 37 nm [33]. Our conventional hydrothermal method provides intermediate sizes of ZnO crystallites.

**Figure 2:**
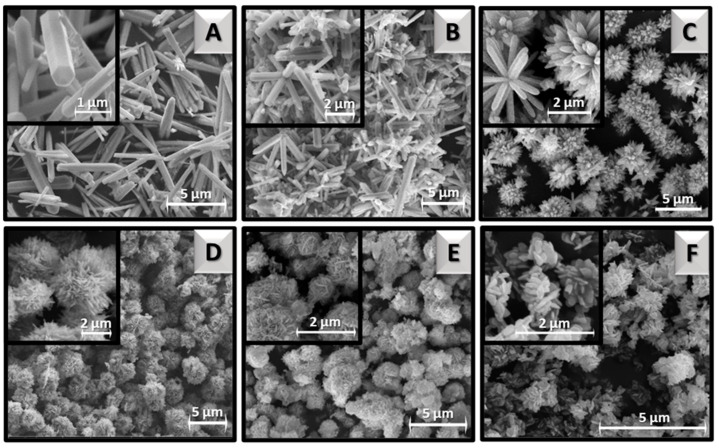
SEM images of ZnO particles synthesized hydrothermally at different temperatures: 100 °C (**A**), 110 °C (**B**), 120 °C (**C**), 160 °C (**D**), 180 °C (**E**), and 200 °C (**F**).

**Figure 3:**
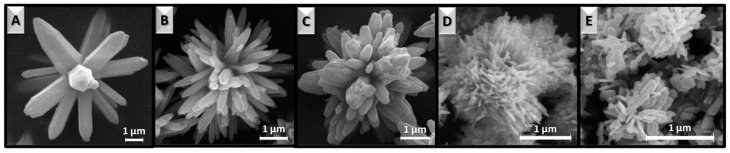
SEM images of ZnO*_x_* flower-like superstructures: ZnO_110_ (**A**), ZnO_120_ (**B**), ZnO_160_ (**C**), ZnO_180_ (**D**), and ZnO_200_ (**E**), where *x* subscripts indicate the exact temperature (°C) of hydrothermal synthesis.

All synthesized ZnO samples are in the form of white, fine-crystalline powders, which differ in morphology and size under the scanning electron microscope. The shapes of material particles and the transformation specifically of the flower-like structures are presented in Figure 2 and Figure 3, respectively. At the lowest temperature (100 °C), distinctly separated hexagonal rods were synthesized (Figure 2A). Their thickness is <500 nm, and their length reaches up to ~5 μm. Interestingly, increasing the temperature to 110 °C resulted in the agglomeration of the rods (Figure 2B) into a flower-like shape. The center of the 3D structures is the core of the flower, consisting of several joined rods of nanometer diameter, from which the rod-like petals radially diverge (Figure 3A). Lai et al. achieved a very similar shape in their study by additionally using ultrasonic processing in the synthesis [23]. Even more similar morphology to the flower structure was obtained at 120 °C (Figure 2C), where more petals with shorter lengths and predominantly pointed tips (sword-like) were synthesized (Figure 3B). The ZnO_120_ sample, among others, is characterized by high homogeneity, which is unique for such complex structures. The ZnO_160_ sample also exhibits homogeneity but it did not develop petals with typical rod morphology (Figure 2D). Many more petals have been formed, which are shorter (~2 μm) and less uniform in shape resembling roses of size ~4 μm (Figure 3C). Increasing the temperature to 180 °C resulted in typical rose formation (Figure 2E and Figure 3D) but smaller in size (~3 μm). Each rose is composed of thin plates that intersect at different angles. Due to a large number of potential petals, it is difficult to distinguish the core of the flower. At even higher temperatures, i.e., 200 °C, rosettes are smaller (Figure 2F), which is probably the reason for the degradation of citric acid or citrate complexes in the system. This is most evident at the highest temperature, where the structures have disintegrated, resulting in smaller irregular plate-like particles of nanometer size (Figure 3E).

**Figure 4:**
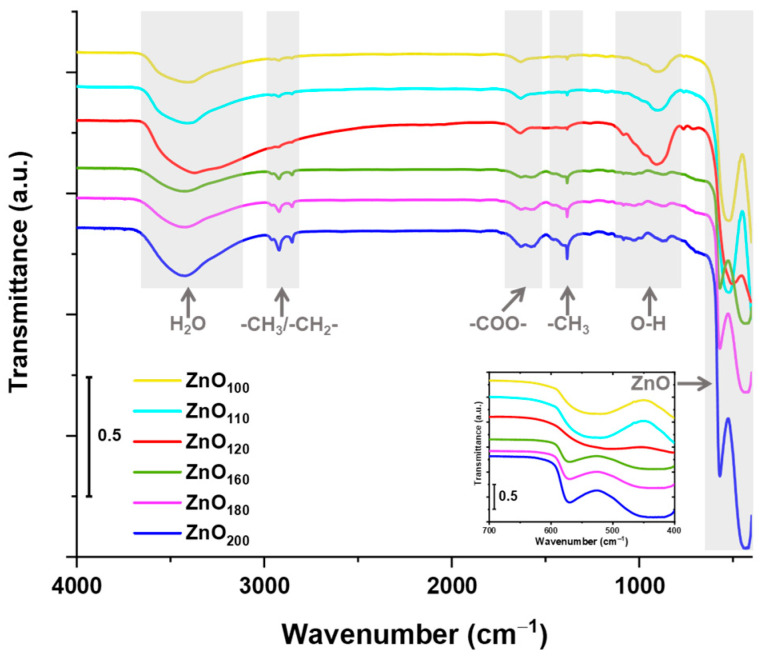
FT-IR spectra of ZnO*_x_* particles synthesized hydrothermally at a temperature in the range of 100–200 °C, where *x* subscripts indicate the exact temperature of synthesis.

Regardless of the XRD profiles of the samples indicating high ZnO phase purity, the detection limit of the method may not have identified compounds additionally formed in the reaction with citric acid. Therefore, FT-IR spectra were acquired, which are depicted in Figure 4. For all samples, bands at 620–400 cm^−1^ were noted, which are characteristic for metal–oxygen bonds [34]. At a wavenumber of 403 cm^−1^, a band attributed to Zn–O, can be observed [23]. However, for ZnO_100_, ZnO_110_, ZnO_160_, ZnO_180_, and ZnO_200_ samples, a band at ~570 cm^−1^ originating from Zn–OH bond is also prominent [35]. It can be suggested that at lower temperatures, residues of non-transformed Zn(OH)_2_ are present, which are least pronounced in ZnO_120_ FT-IR spectrum. At higher temperatures, the band attributed to Zn–OH derives from the formation of zinc citrate and hydroxide complexes. Materials’ spectra also indicated the broad band in the ~3650–3200 cm^−1^ range corresponding to the adsorbed water. At 2960, 2920, and 2851 cm^−1^, bands are indicative of symmetric and asymmetric C–H bonds. The bands present at the 1632–1571 cm^−1^ range correspond to the carboxylate (–COO–) asymmetric and symmetric stretching vibrations, which signifies ZnO–CA complexes were obtained. Other bands were noted at 1410 cm^−1^ and 1386 cm^−1^, suggesting the ZnO particles were enriched with aliphatic- or carbohydrate-OH functionalities [36]. The aforementioned bands showed increasing intensity with the rising temperature of synthesis, which may imply that the higher temperature more strongly contributed to the incorporation of citric acid into the structure of ZnO. Interestingly, the opposite trend in intensity is shown by the band in the 1042–812 cm^−1^ range assigned to O–H bending vibration, which is more intense for the samples synthesized at lower temperature ranges (100–110 °C). This band could be more likely assigned to Zn–OH, which again indicates unreacted hydroxide intermediate product residue in the ZnO material [37]. Hence, it can be concluded that in order to achieve complete substrate conversion and obtain pure ZnO, the reaction should be carried out at min. 120 °C; however, with consideration of the residual citric acid in the material at higher temperatures.

**Figure 5:**
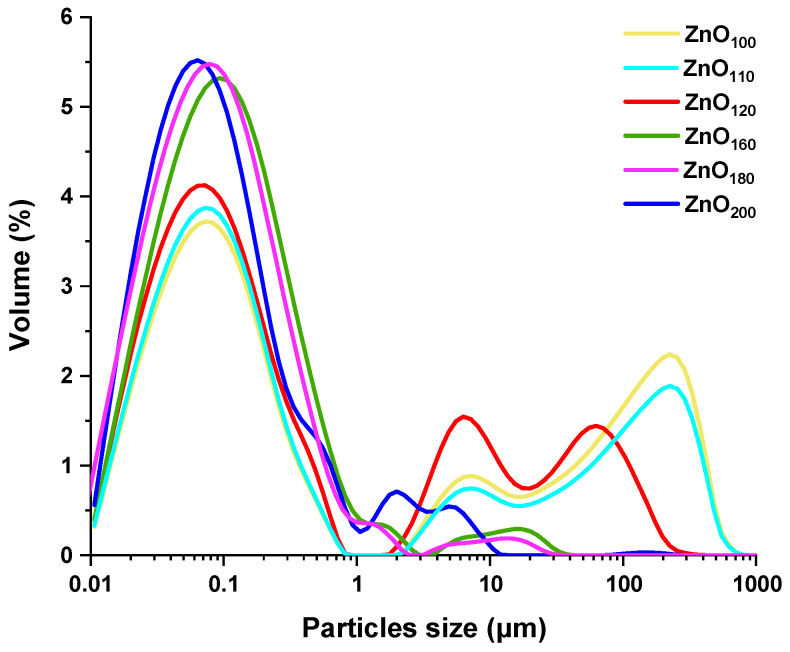
Particle size distribution of ZnO samples synthesized hydrothermally at 100, 110, 120, 160, 180, and 200 °C.

A detailed analysis of the particle size distribution of the obtained materials was carried out by laser diffraction, the results of which are displayed in Figure 5. The course of the particle size distributions of ZnO samples obtained by hydrothermal treatment with citric acid and different temperatures is multimodal. This is mainly due to the elongated shape of the particles, as well as the varying homogeneity of the materials. Among the several peaks for each sample, a predominant one in the range of 0.02 to 1 μm was noted, but with different intensities represented as % volume share, with the maximum value of 5.5%. As the synthesis temperature increased, the proportion of very small particles tended to rise for all samples. The percentage of specific particle sizes in the total sample volume *d*(0.1), *d*(0.5), *d*(0.9), along with De Brouckere’s and Sauters’ mean diameters is shown in Table 2. Analyzing these data, it can be established that as the hydrothermal treatment temperature increases, the median value of the particle distribution size decreases from 0.182 μm to 0.076 μm. The highest proportion of large particles is found in the case of ZnO obtained at 100 °C, where as much as 90% of the particle distribution is smaller than 209 μm. In the case of a large volume proportion of small particles, the best results were obtained when synthesized at 200 °C, as 10% of ZnO particles are up to 0.022 μm in size, and 90% have diameters below 0.557 μm. A consistent decline in values is also observed for the average particle diameter D[3,2], decreasing from 0.084 μm to 0.053 μm. A significant reduction was observed for particle size distribution weighted by volume D[4,3]. For ZnO_100_, ZnO_110_, and ZnO_120_ samples, the values accordingly are: 52.000 μm, 37.600 μm, and 12.300 μm, however, the use of high temperatures from 160 °C upwards resulted in sizes oscillating at ~0.576 ± 0.020 μm. The larger sizes for materials obtained at 100–120 °C indicate their greater tendency to agglomeration caused by rod-like shape. Higher synthesis temperatures confirm the intensification of reagent diffusion and the prevention of long-range crystallization into larger microstructures.

**Figure 6:**
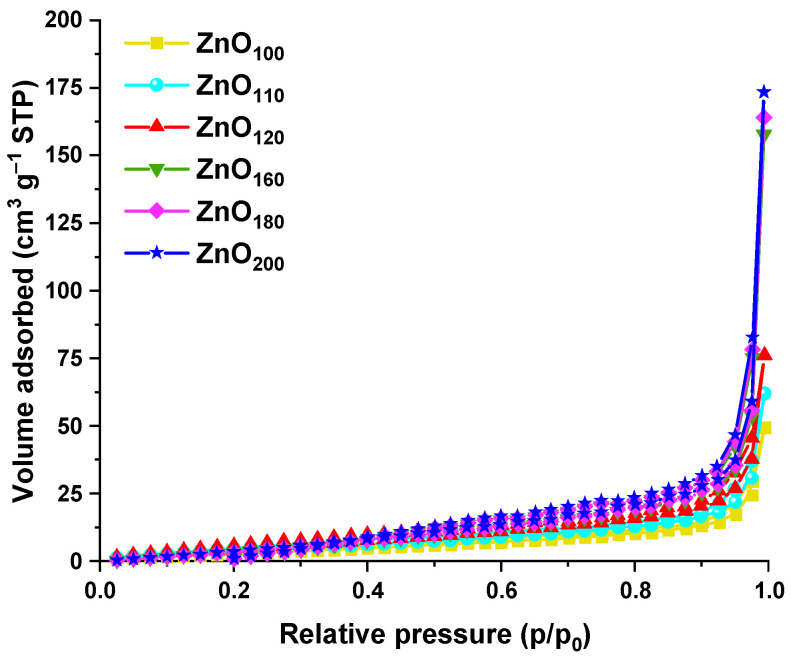
Nitrogen adsorption/desorption isotherms of ZnO*_x_* samples, synthesized at various temperatures (100–200 °C), where *x* subscripts indicate the exact thermal conditions of synthesis.

Nitrogen physisorption was carried out for all synthesized samples, and the adsorption isotherms obtained are presented in Figure 6. The materials after syntheses over the entire temperature range revealed a similar course of isotherms according to IUPAC classification as type IV. In addition, the H3 type of hysteresis loops is noted at *p*/*p*_0_ > 0.8 [37]. On this basis, the mesoporosity of the materials can be ascertained, which is an outcome of the synthesis course with citric acid similar to the templating method. The detailed textural parameters such as specific surface area, total pore volume and average pore diameter are collected in Table 3. The materials demonstrated S_BET_ in the range of 10–37 m^2^ g^−1^ typical of ZnO. The lowest specific surface areas, i.e., 10, 12, and 18 m^2^ g^−1^ displayed ZnO_100_, ZnO_110_, and ZnO_120_ samples, respectively. This is due to diminished diffusion of hydroxide and citrate complexes at lower temperatures (100–120 °C). Materials with lower specific surface areas are characterized by hexagonal and rod-like shapes with smooth surfaces. Effective crystallization of defect-free crystalline superstructures contributed to poorer porosity and smaller total pore volumes. In turn, increasing the temperature to 160 °C resulted in a more than two-fold higher developed specific surface area of 37 m^2^ g^−1^ and pore volume of 0.24 cm^3^ g^−1^, suggesting a significant influence of the diffusion of the reagents on the porosity induced by the capping agent. The increasing temperature even further led to a disorder of the dispersion system, partial degradation of CA, and S_BET_ development to 27 m^2^ g^−1^ and 25 m^2^ g^−1^, for ZnO_180_ and ZnO_200_ samples, respectively. More developed surface area for rose-like superstructures and more pronounced hysteresis loops in isotherms above *p*/*p*_0_ = 0.8 for ZnO_160_, ZnO_180,_ and ZnO_200_ indicate the interparticle porosity [38]. Nanostructured petals radiating from the center of each structure are packed in such a way that they have additional spaces, which increase the specific surface area.

By means of the abovementioned findings, we proposed the course of processes that could take place in the reaction system. Citric acid as a capping agent complexes the Zn^2+^ ions, ensuring their dispersion in solution and creating colloidal clusters. Such systems provide a soft template for ZnO formation. However, it is a temporary assembly, because during the hydrothermal process in the presence of OH^−^ the formation of ZnO crystallites takes place within the clusters. Consequently, a hard template-directing process occurs, where clusters take over the role of the template [23]. Moreover, citrates can further react with the newly created ZnO and attach to it, as they contribute to its partial dissolution (1) along with the dispersion/stabilization of Zn^2+^ [39]. The course of crystallization is also affected by the interaction of OH^−^ with CA. [Zn(OH)_4_]^2−^ complexes serve as growth units, which by dehydration via heating form ZnO crystallites (2−4) [40]. In the presence of CA, they can react with it (5, 6) but also [Zn(C_6_H_5_O_7_)_4_]^10−^ can be formed, which may contribute to an insufficient amount of the [Zn(OH)_4_]^2−^ complex [41].

3 ZnO_(*s*)_ + 2 C_6_H_8_O_7(*s*)_ → 3 Zn^2+^_(*aq*)_ + 2 C_6_H_5_O_7_^3−^_(*aq*)_ + 3 H_2_O_(*l*)_ (2)

Zn^2+^ + 2 OH^−^ ↔ Zn(OH)_2_ (3)

Zn(OH)_2_ + 2 OH^−^ ↔ [Zn(OH)_4_]^2−^ (4)

[Zn(OH)_4_]^2−^ → ZnO + H_2_O + 2 OH^−^ (5)

Zn(OH)_2(*s*)_ + C_6_H_5_O_7_^3−^_(*aq*)_ ↔ Zn(C_6_H_5_O_7_)^−^_(*ads*)_ + 2 OH^−^_(*aq*)_ (6)

Zn(C_6_H_5_O_7_)^−^ _(*ads*)_ ↔ [Zn(C_6_H_5_O_7_)^−^]_(*aq*)_ (7)

**Figure 7:**
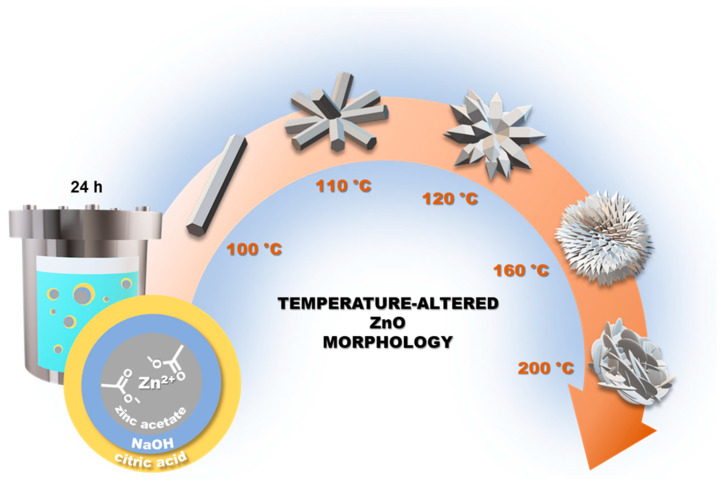
Scheme of temperature-altered (100–200 °C) hydrothermal synthesis of ZnO, resulting in rod-, flower-, and rose-like microstructures.

In our case, a generous volume of alkali solution was used, providing a sufficient amount of hydroxyl anions. Therefore, the course of ZnO crystallization was mainly relying on the intensity of temperature-dependent diffusion of citrates, as well as the stability of the citrate complexes, which could decline at high temperatures. ZnO nanocrystals have hexagonal packing, which itself increases anisotropy due to the associated internal dipole moment along the main axis of the crystal lattice. Moreover, the partial polar characteristic and anisotropy of the ZnO crystal structure promote the growth of nanorods [42]. The most polar surface of crystallites is the basal plane (0 0 1), whereas the anisotropic growth occurs along the c-axis in the [0002] direction [43]. It is caused by the moderately positive Zn lattice points of one end of the basal polar plane and negative oxygen lattice points on another. Using a capping agent and chelating Zn^2+^ can impede crystal growth and the formation of isolated rods can be impaired. A schematic representation of the temperature-dependent morphological transformations of ZnO is depicted in Figure 7. At a temperature of 100 °C, no citric acid effect was noted and conventional rod growth was observed. This may be due to the poor diffusion of Zn^2+^ and insufficient amount of CA for their full complexation. In earlier reports on the synthesis with CA, sonication was found to have a significant effect on the orientation of crystallization. Its application contributed to the formation of more flower-like structures with well-defined petals [23]. In our case, the temperature was responsible for this effect. Temperature higher than the boiling point of water, i.e., 110 °C, significantly altered the diffusion of cations, conditioning multidirectional crystallization. The next reason for obtaining flower-like structures is that rods have a higher tendency to agglomerate through lower activation energy than spheres and greater attraction by van der Waals forces [44]. Increasing the temperature from 100 to 110 °C led to the merging of rod-like crystallites into flowers and a slightly higher temperature of 120 °C intensified the formation of petals, at the expense of their length. The higher the temperature, the greater the diffusion, which prevented the formation of hexagonal structures. At 160 °C and 180 °C, the Zn-terminated growing face was highly capped with citric acid, hindering its further growth along face orientation [45]. As a result, planar flake-like structures were obtained. The highest temperature of 200 °C led to the formation of the least regular microstructures with the smallest particles. This may be the effect of preventing steady growth and the degradation of citric acid.

### 3.2. Characterization of ZnO Particles Obtained via pH-Altered Hydrothermal Method

**Figure 8:**
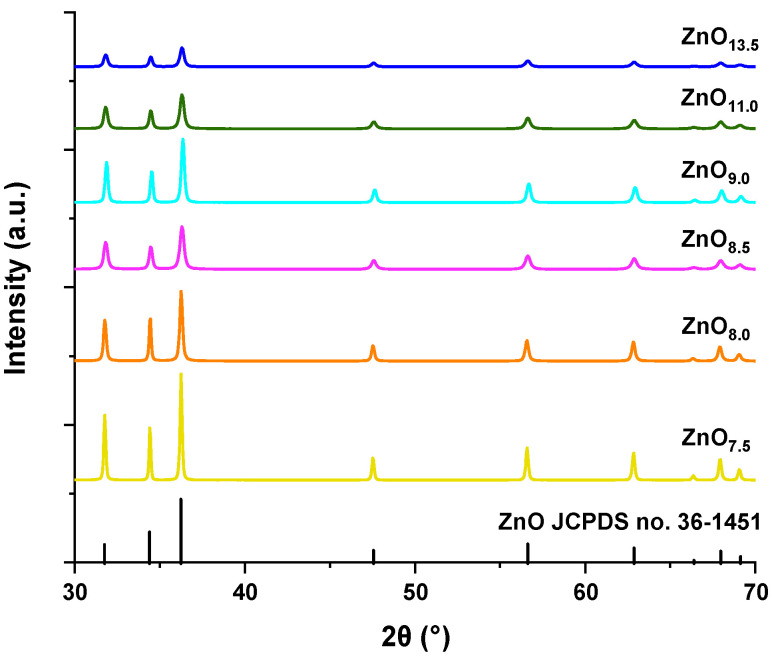
X-ray diffraction patterns of ZnO particles synthesized hydrothermally at different pH: 7.5, 8.0, 8.5, 9.0, 11.0, and 13.5. All compared to the standard ZnO, polycrystalline hexagonal wurtzite (JCPDS card No. 36-1451).

It has already been confirmed in earlier reports that the pH of the reaction solution has a strong impact on the shape of ZnO [4]. Wahab et al. fabricated sheet-shaped ZnO at pH 6, while at strongly alkaline pH (10–12) material’s morphology changed to nanorods. The pH did not significantly influence the crystallinity of the samples [46]. However, the use of different pH in our case contributed to obtaining materials with different morphology as well as a degree of crystallinity. ZnO particles synthesized were subjected to XRD analysis (Figure 8). It was noted that in each case pure-phase zinc oxides were obtained. The peaks present in the diffractograms correspond to planes consistent with the polycrystalline hexagonal ZnO wurtzite reference. Depending on the pH used, reflections of different intensities were observed for individual samples. The most crystalline sample was ZnO_7.5_ obtained at the lowest pH, which indicated the sharpest reflections. Increasing the pH resulted in lower peak intensities for ZnO_8.0_ and ZnO_8.5_ materials. The reflections also become wider, which may indicate the appearance of smaller crystallites. The adjustment of the NaOH solution amount in order to reach pH 9.0 resulted in higher crystallinity, whereas strongly basic conditions of pH 11.0 and 13.5, successively lowered the crystallinity. The ZnO_13.5_ sample exhibited the lowest degree of crystallinity among all materials. In addition, the pH of the solution was found to affect the size of the crystallites (Table 4). A strongly alkaline environment with a pH of 13.5 led to the synthesis of ZnO crystallites with a size of 18.98 nm, while the closest to the neutral environment (pH 7.5) yielded the largest crystallites with a size of 59.22 nm. Kubiak et al. obtained ZnO crystallites decreasing in size from 48.3 nm to 24.3 nm in the pH range of 8.0–12.0 using microwave-assisted synthesis [29]. In our case, an initial decrease in the size of the crystallites was observed up to pH 8.5 (33.67 nm), and at pH 9.0 the crystallites become larger (41.10 nm), and then they decreased again under more alkaline conditions. This may be an effect of the synthesis method consisting of two steps. The first one included NaOH solution dropping, crystallization, and precipitation of Zn(OH)_2_ complexes, followed by a hydrothermal treatment without calcination.

**Figure 9:**
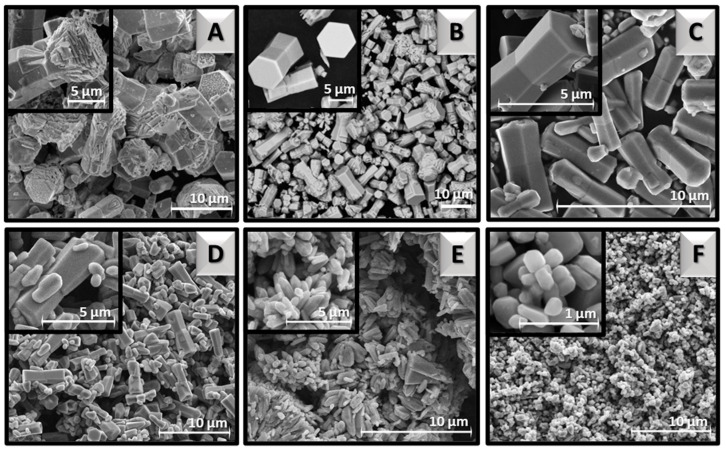
SEM images of ZnO particles synthesized hydrothermally at different pH: 7.5 (**A**), 8.0 (**B**), 8.5 (**C**), 9.0 (**D**), 11.0 (**E**), and 13.5 (**F**).

ZnO synthesis without capping agents caused the formation of different morphologies displayed in Figure 9, that resemble hexagonal pellets, alternatively termed dog-bones. SEM images present the successively changing shape of the ZnO particles as the pH was adjusted in the range of 7.5–13.5. The lack of application of CA along with a large amount of NaOH solution at first led to pH 13.5, which was then successively reduced by lowering the amount of alkali volume. Subsequent process conditions at a high temperature of 160 °C contributed to the formation of nanometric prismatic crystallites (Figure 9F). They indicated a length of up to 500 nm and a thickness of up to 200 nm. The high pH favored their growth, hence it was successively lowered to achieve homogeneous microstructures. The pellets started to form at pH 9.0 (Figure 9D), but they were still covered with irregular particles on the surface. More interestingly, a slightly lower pH, especially 8.5 (Figure 9C) provided more favorable conditions for the crystallization of the homogeneous short rod-like structures with a length of 5 μm, and thickness of ~2 μm. Too low pH, i.e., 7.5 (Figure 9A), led to the formation of hexagonal structures, however, with a large number of defects and cavities.

**Figure 10:**
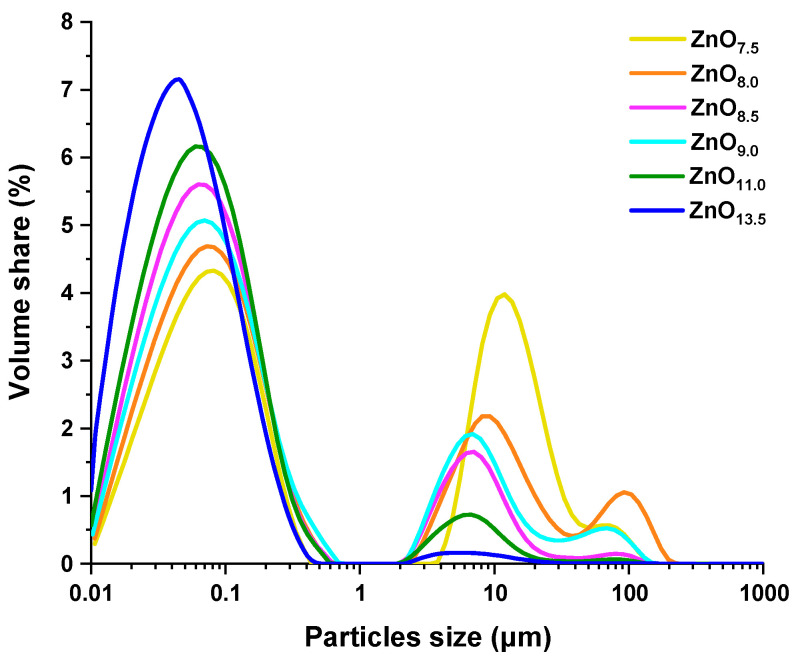
Particle size distribution of ZnO samples synthesized hydrothermally at pH 7.5, 8.0, 8.5, 9.0, 11.0, and 13.5.

Particle size distribution curves have mostly a multimodal course, which consists of three distinct peaks (Figure 10). The percentages of specific particle sizes in the total sample volume *d*(0.1), *d*(0.5), and *d*(0.9) are included in Table 5. The data show that the pH of the reaction environment affected the distribution and particle size of the materials. Although 10% of the ZnO particle distribution for all syntheses have similar sizes within the range of 0.022–0.029 μm, a significant difference occurs at the parameter *d*(0.9). The highest proportion of small particles was observed for ZnO_11.0_ and ZnO_13.5_, for which 90% of the particles are smaller than 0.687 μm and 0.568 μm, respectively. In contrast, ZnO synthesized at pH 7.5 showed the lowest proportion of small particles. Interestingly, the values of the D[4,3] parameter show that there is no linear relation between the size of the ZnO particles and the pH of the reaction medium. It indicated that the material prepared at the highest pH (13.5) has the smallest volume mean diameter, i.e., 1.500 μm, and that at the lowest pH (7.5) has D[4,3] of 7.580 μm. At pH 8.0 the largest particles with a mean diameter of 9.240 μm were prepared. Nonetheless, the particle size distribution for highly alkaline conditions has a monomodal course, with the largest contribution of small-sized particles.

**Figure 11:**
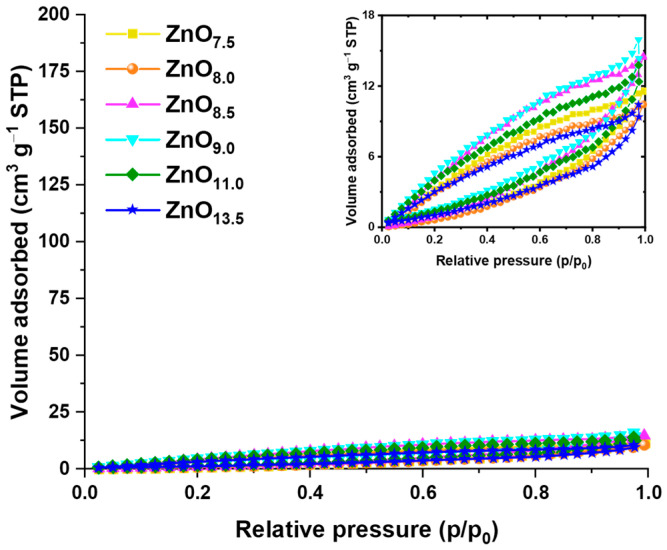
Nitrogen adsorption/desorption isotherms of ZnO*_x_* samples, synthesized in various pH (7.5–13.5), where *x* subscripts indicate the exact pH reaction conditions.

Adsorption/desorption of nitrogen performed for ZnO materials revealed their poor porosity. Samples indicated adsorption isotherms (Figure 11) qualifying for type II according to IUPAC classification, typical of non-porous materials [47]. This is likely due to the absence of a capping or pore-forming agent. The scope of the obtained specific surface areas is in the range of 9–21 m^2^ g^−1^, where the highest surface area was observed for ZnO_9.0_ material, and the lowest for ZnO_13.5_. Other samples displayed the following S_BET_ values: ZnO_7.5_–14 m^2^ g^−1^, ZnO_8.0_–10 m^2^ g^−1^, ZnO_8.5_–12 m^2^ g^−1^, and ZnO_11.0_–16 m^2^ g^−1^. Samples with larger porosity development may be the result of defects formed, or agglomeration of crystallites onto hexagonal pellets, which contributed to the formation of additional spaces between the material particles. Interestingly, a very high pH of 13.5 and the resulting nanometric prisms are characterized by the lowest surface development, due to the lack of formation of flake nanostructures and the preservation of smooth, non-porous surfaces. This is comparable to the ZnO_8.0_ sample of S_BET_ = 10 m^2^ g^−1^, which, despite having larger hexagonal pellet particles, has perfectly smooth surfaces of crystallized oxide superstructures.

**Figure 12:**
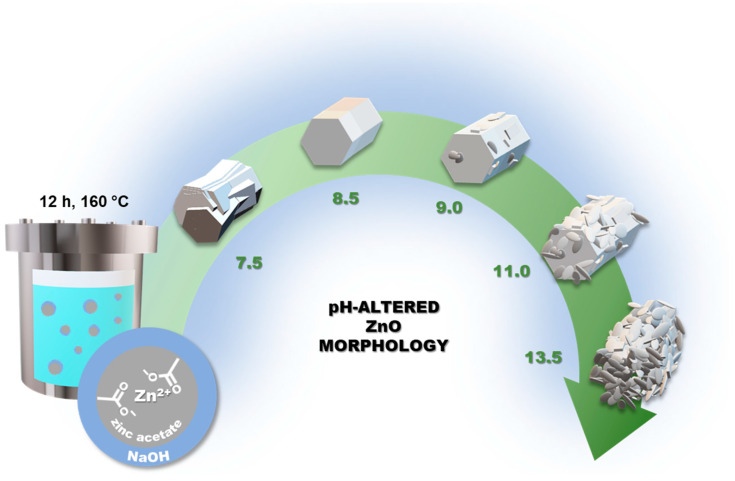
Scheme of pH-altered (7.5–13.5) ZnO hydrothermal synthesis, resulting in hexagonal pellets and plate-like particles.

Zhang et al. by regulating the pH of the solution without using any capping agent in the hydrothermal synthesis obtained morphologies entirely different from our results [48]. Despite utilizing similar reactants, i.e., zinc acetate, and NaOH solution, as well as similar high pH values of the reaction mixtures (12.0 and 13.5), they synthesized anisotropic particles at 120 and 160 °C, and flowers at 200 °C. Hence, it can be concluded that ZnO shape regulation is a much more complex phenomenon, often dependent on many factors, and sometimes on the mere initialization of crystallization and sustained provision of [Zn(OH)_4_]^2−^ complexes. A proposal for the reaction in the system without organic compound additive is shown below. At first, the formation of Zn(OH)_2_ occurs (7), followed by the generation of a building block complex in the presence of water (8). Next, depending on the offset of the equilibrium state by external factors complex is converted to ZnO (9, 10). This happened in our hexagonal pellets synthesis, in which a NaOH solution was added very slowly toward a specific pH adjustment (Figure 12). Continuous stirring provided sufficient hydroxide complexes diffusion for the formation of short hexagonal pellets, however, without signs of multidirectional crystallization. The lack of elongated rods indicates that both the slow addition of alkali solution and OH^−^ generation, inhibited their development. A ZnO_7.5_ sample with a large number of defects indicates that a pH of 7.5 is too low with a small amount of OH^−^ to form plain superstructures. The most optimal conditions were 8.0 and 8.5, where most homogeneous hexagonal pellets have been achieved. Further addition of alkali led to the formation of irregular crystallites on the pellet surface. Moreover, the high pH caused a complete loss of superstructure morphology, which could be due to the formation of a large number of crystallization nuclei in the system without enabling their steady growth. This demonstrates that it is possible to obtain a certain morphology of ZnO without using organic additives such as CA, and only a simple pH adjustment with a slow provision of alkali solution is sufficient.

Zn(CH_3_COO)_2_·2H_2_O + 2NaOH → Zn(OH)_2_ + 2CH_3_COONa + 2H_2_O (8)

Zn(OH)_2_ + 2OH^−^ → [Zn(OH)_4_]^2−^ (9)

[Zn(OH)_4_]^2−^ ↔ ZnO_2_^2−^ + 2H_2_O (10)

ZnO_2_^2−^ + H_2_O ↔ ZnO + 2OH^−^ (11)

Zinc oxide, featuring a variety of morphological forms, can be successfully prepared as flowers of varying petal sizes merely by adjusting the reaction temperature. In this case, it is possible to influence the kinetics of zinc cation complexation and the degree of diffusion of hydroxide/citrate complex systems, and further the direction of crystallization with the formation of porous zinc oxide superstructures. It was necessary to use citric acid as a capping agent for this process, which was essential to induce the direction of crystallization of long rods with a greater tendency to agglomerate and form radial structures. However, if one omits the citric acid and uses pH adjustment it is feasible to regulate the shape, homogeneity, and size of hexagonal ZnO pellets. This is determined by the amount and dropping manner of NaOH solution, followed by hydroxyl anions generation to form zinc complexes as the building blocks for ZnO.

## 4. Conclusions

Well-defined zinc oxide morphologies were obtained by a facile hydrothermal synthesis in which the temperature range (100–200 °C), as well as pH (7.5–13.5), were optimized. In the case of using a capping agent such as citric acid, it was found that the temperature and consequently the diffusion of hydroxide and citrate complexes must be optimized to obtain the desired ZnO morphology. Too low temperature (100 °C) of reaction ensures slow crystallization resulting in larger and typical ZnO structures, meaning conventional hexagonal rods. An intermediate temperature provides sufficient dispersion of reagents toward yielding complex microstructures. It was shown that it requires an auxiliary suitable temperature, (110–160 °C), to obtain materials with a shape typical of flowers. High temperatures such as 180 °C and 200 °C lead to the formation of petal-like irregular nanostructures via the degradation of citrates, which also can serve as an agent generating porosity. Moreover, it was shown that without using any capping agent, it is possible to produce ZnO with high homogeneity. It was caused by a slow dropping of NaOH solution, as well as a pH of 8.0–8.5, at which hexagonal pellets were obtained. A strongly alkaline environment of pH 11.0 and 13.5 favors the formation of nanosized particles without ordered superstructures. The materials presented have been obtained as one of the precursors for MOF synthesis. The development of ZnO preparation aimed to yield a unique source of cations for metal nodes in the construction of porous scaffolds. ZnO with the potential to facilitate morphology control by means of templating effects and the ready availability of deposition techniques on metal oxides are an advantage for gaining control over the spatial localization of the MOF crystallization.

## Figures and Tables

**Table 1:** ZnO*_x_* crystallite sizes estimated via Scherrer’s equation for the plane (1 0 1). Subscript *x* corresponds to the temperature (°C) of the hydrothermal synthesis.

Material	Peak Position 2θ (°)	Crystallite Size (nm)
ZnO_100_	36.32	16.80
ZnO_110_	36.32	17.18
ZnO_120_	36.29	20.41
ZnO_160_	36.36	36.87
ZnO_180_	36.25	48.36
ZnO_200_	36.25	56.15

**Table 2:** Particle size distribution data of ZnO*_x_* synthesized at various temperatures (100–200 °C), where *x* subscripts indicate the exact reaction conditions.

Material	D[3,2] (μm)	D[4,3] (μm)	*d*(0.1) (μm)	*d*(0.5) (μm)	*d*(0.9) (μm)
ZnO_100_	0.084	52.000	0.029	0.182	209.000
ZnO_110_	0.079	37.600	0.026	0.153	113.090
ZnO_120_	0.072	12.300	0.026	0.131	46.800
ZnO_160_	0.062	0.556	0.025	0.098	0.460
ZnO_180_	0.060	0.581	0.024	0.081	0.509
ZnO_200_	0.053	0.590	0.022	0.076	0.557

**Table 3:** Textural parameters of ZnO synthesized in the temperature range of 100–200 °C.

Material	BET Surface Area (m^2^ g^−1^)	Total Pore Volume (cm^3^ g^−1^)	Average Pore Diameter (nm)
ZnO_100_	10	0.04	23.43
ZnO_110_	12	0.06	20.08
ZnO_120_	18	0.10	22.13
ZnO_160_	37	0.24	26.22
ZnO_180_	27	0.16	21.45
ZnO_200_	25	0.14	22.67

**Table 4:** ZnO*_x_* crystallites sizes estimated via Scherrer’s equation. Subscript *x* corresponds to the pH adjusted before the hydrothermal synthesis.

Material	Peak Position 2θ (°)	Crystallite Size (nm)
ZnO_7.5_	36.34	59.22
ZnO_8.0_	36.27	49.03
ZnO_8.5_	36.27	33.67
ZnO_9.0_	36.41	41.10
ZnO_11.0_	36.27	27.42
ZnO_13.5_	36.33	18.98

**Table 5:** Particle size distribution data of ZnO*_x_* synthesized at various pH (7.5–13.5) of reaction, where *x* subscripts indicate the exact pH reaction conditions.

Material	D[3,2] (μm)	D[4,3] (μm)	*d*(0.1) (μm)	*d*(0.5) (μm)	*d*(0.9) (μm)
ZnO_7.5_	0.081	7.580	0.029	0.142	20.500
ZnO_8.0_	0.069	9.240	0.026	0.112	19.800
ZnO_8.5_	0.055	4.310	0.023	0.079	5.890
ZnO_9.0_	0.062	1.980	0.024	0.095	6.330
ZnO_11.0_	0.055	1.750	0.022	0.082	0.687
ZnO_13.5_	0.055	1.500	0.022	0.083	0.568

## Data Availability

The data presented in this study are available on request from the corresponding author.
